# Plin4-Dependent Lipid Droplets Hamper Neuronal Mitophagy in the MPTP/p-Induced Mouse Model of Parkinson’s Disease

**DOI:** 10.3389/fnins.2018.00397

**Published:** 2018-06-18

**Authors:** Xiaojuan Han, Jialei Zhu, Xinlei Zhang, Qiqi Song, Jianhua Ding, Ming Lu, Sifan Sun, Gang Hu

**Affiliations:** ^1^Department of Pharmacology, School of Medicine and Life Sciences, Nanjing University of Chinese Medicine, Nanjing, China; ^2^Department of Traditional Chinese Medicine, The Affiliated Drum Tower Hospital of Nanjing University Medical School, Nanjing, China; ^3^Jiangsu Key Laboratory of Neurodegeneration, Department of Pharmacology, Nanjing Medical University, Nanjing, China; ^4^Affiliated Hospital of Nanjing University of Chinese Medicine, Nanjing, China

**Keywords:** Plin4, lipid droplets, Parkinson’s disease, DA neurons, mitophagy

## Abstract

Epidemiological studies have shown that both lipid metabolism disorder and mitochondrial dysfunction are correlated with the pathogenesis of neurodegenerative diseases (NDDs), including Parkinson’s disease (PD). Emerging evidence suggests that deposition of intracellular lipid droplets (LDs) participates in lipotoxicity and precedes neurodegeneration. Perilipin family members were recognized to facilitate LD movement and cellular signaling interactions. However, the direct interaction between Perilipin-regulated LD deposition and mitochondrial dysfunction in dopaminergic (DA) neurons remains obscure. Here, we demonstrate a novel type of lipid dysregulation involved in PD progression as evidenced by upregulated expression of Plin4 (a coating protein and regulator of LDs), and increased intracellular LD deposition that correlated with the loss of TH-ir (Tyrosine hydroxylase-immunoreactive) neurons in the MPTP/p-induced PD model mouse mesencephalon. Further, *in vitro* experiments showed that inhibition of LD storage by downregulating Plin4 promoted survival of SH-SY5Y cells. Mechanistically, reduced LD storage restored autophagy, leading to alleviation of mitochondrial damage, which in turn promoted cell survival. Moreover, the parkin-poly-Ub-p62 pathway was involved in this Plin4/LD-induced inhibition of mitophagy. These findings were further confirmed in primary cultures of DA-nergic neurons, in which autophagy inhibitor treatment significantly countermanded the ameliorations conferred by Plin4 silencing. Collectively, these experiments demonstrate that a dysfunctional Plin4/LD/mitophagy axis is involved in PD pathology and suggest Plin4-LDs as a potential biomarker as well as therapeutic strategy for PD.

## Introduction

Parkinson’s disease (PD) is a progressive neurodegenerative disorder characterized by the preferential loss of dopaminergic neurons in the SNpc, affecting 2–3% of the population over the age of 65 (Poewe et al., 2017). The exact etiology and natural course of PD have yet to be fully clarified but involve dysfunction of numerous system-level processes, including mitochondrial function, calcium and dopamine homeostasis, neuroinflammation, and autophagy (Athauda and Foltynie, 2015; Ascherio and Schwarzschild, 2016; Poewe et al., 2017), highlighting the predominant role of mitochondrial dysfunction (Ryan et al., 2015; Onyango et al., 2017). Genome-wide association studies (GWAS) have identified many of the PD-associated genes such as PINK, PARKIN and DJ-1, which have been shown to either directly or indirectly play roles in mitochondrial homeostasis or mitophagy (Dawson and Dawson, 2017; Onyango et al., 2017). Moreover, recent studies have indicated that abolished mitophagy-mediated clearance of mtDNA and mtROS rendered impaired mitochondria as activators of the NLRP3 inflammasome to trigger neuroinflammation and promote DA neuronal loss (Yan et al., 2015; Zhong et al., 2016). Thus, excessive mitochondrial stress in response to genetic or environmental toxins may result in the death of neurons, and the capability to remove damaged mitochondria through mitophagy must be well controlled. Thus, the role that regulation of mitophagy plays in the pathogenesis of PD needs further exploration.

Recent reports revealed that LD-related lipotoxicity might participate in PD pathology (Wang et al., 2002; Outeiro and Lindquist, 2003; Schaffer, 2016). LDs are highly dynamic organelles that emerge from the ER membrane and serve as the intracellular sites for neutral lipid storage (Hashemi and Goodman, 2015). Accumulating evidence suggests that LDs play a much broader role in biology than previously indicated, including sequestering transcription factors, generating ligands for nuclear receptors, and regulating immunity (Welte, 2015). As to NDDs, α-synuclein, a pathogenic protein in PD, was reported to bind to LDs *in vitro* (Thiam et al., 2013), and subsequent *in vivo* studies confirmed that aggregated LDs in glia accelerated neurodegeneration in Drosophila (Bailey et al., 2015; Liu et al., 2015). Combined, these studies suggest that an LD abnormality may contribute to NDDs. Mechanistically, emerging evidence reveals an unexpected intimate association between LDs and other intracellular organelles, especially the ER, autophagic lysosomes and mitochondria, to affect their functions (Jaishy and Abel, 2016). Notably, LDs were reported to affect mitochondrial fusion dynamics, ensuring maximum oxidative metabolism and homeostasis (Rambold et al., 2015). Thus, the precise modulation of LDs is maintained to guarantee mitochondrial quality control.

Perilipin family members (Plin1-5), the surrounding proteins of LDs, are regarded as the most important regulator of LDs, facilitating LD movement and cellular signaling interactions (Kimmel and Sztalryd, 2016). Among them, Plin2 has been widely studied and proposed as a target for counteracting both metabolic and age-related diseases (Conte et al., 2016). Plin4 (S3-12), a perilipin normally expressed in heart and skeletal muscle, was reported to be involved in cardiac lipid accumulation (Chen et al., 2013). These studies collectively support the idea that perilipins may play a common role in pathological degenerative conditions. Although a few reports have indicated a relationship between LDs and α-synuclein or mitochondria, the detailed involvement and underlying mechanism of perilipin-regulated LDs in the pathogenesis of PD are not yet clarified.

In this study, using both cell and animal models and a combination of RNA-seq and LD-specific indicators, we illustrate that the Plin4/LD/mitophagy axis has a crucial role in neurodegeneration resulting from MPTP/MPP^++^ insult and indicate Plin4-LDs as a potential biomarker as well as therapeutic strategy for PD.

## Materials and Methods

### Mice

**FIGURE 1:**
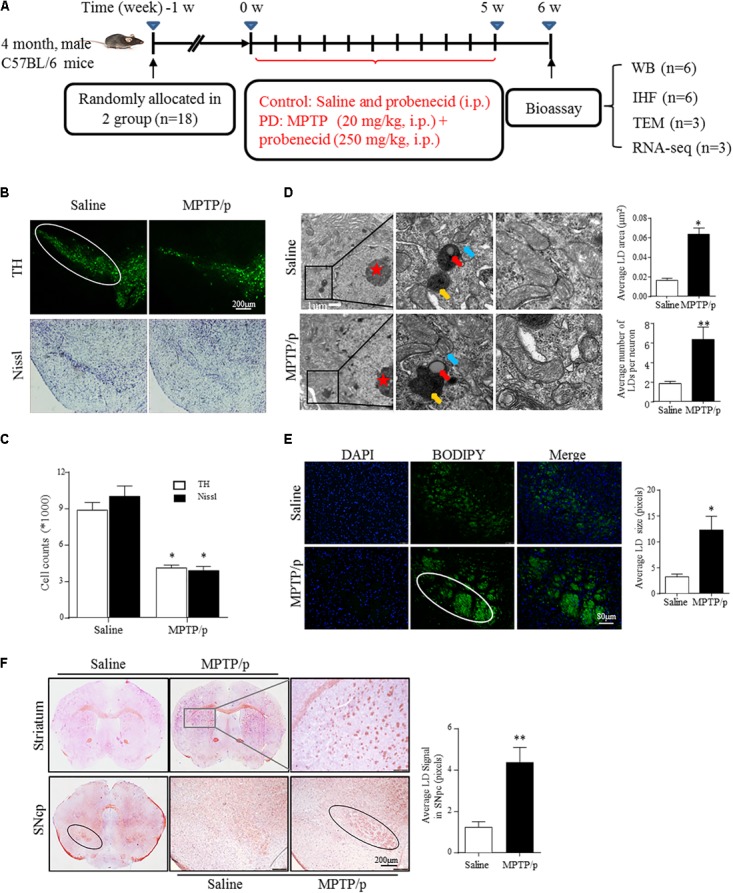
Lipid droplets (LDs) accumulate in the mesencephalon of MPTP/p-treated mice. **(A)**: Diagram of the experimental design. Four-month-old male C57BL/6 mice were grouped and treated as indicated. Mice were transcardially perfused at the endpoint for bioassay. **(B,C)** A: TH and Nissl staining of the mouse brain sections. In a saline-injected control, there is a dense TH^+^ and Nissl^+^ network of cell bodies in the SNpc. After MPTP injection, there is a dramatic reduction in TH immunoreactivity and Nissl staining. Scale bar: 200 μm. B: Stereology of the loss of SNpc neurons in NC and MPTP-dosed mice. ^∗^*P* < 0.05 vs. saline group, determined by one-way ANOVA (*n* = 6). **(D)** TEM of mesencephalon tissues comparing LD accumulation in neurons (with red stars indicating the nucleus) and distribution relative to organelles, as indicated. Middle frames with red arrows show lipid containing vesicles, yellow shows the autolysosome and blue shows mitochondria. Right frames displayed normal (up) and abnormal (down) mitochondria. **(E,F)** Tissues from **(B)** stained with BODIPY **(E)** and Oil Red O **(F)**. BODIPY showed more LD accumulation in SNpc (outlined in white) of PD model mice, in contrast to controls. **(F)** Whole-mount brain sections show LD accumulation in the SNpc (outlined in black), and abnormal intensely stained cross-sections of TH^+^ axon morphology can be seen in the striatum (frame shows higher magnification) of PD model mice, in contrast to the controls. Scale bar as indicated. Data shown in **(D–F)**: quantitation of LD number and size. ^∗^*P* < 0.05, ^∗∗^*P* < 0.01 vs. saline group as determined by Student’s *t*-test (*n* = 4).

Male C57BL/6 mice (4 months old) weighing 24–30 g were purchased from the Model Animal Research Center of Nanjing Medical University (Nanjing, China). All mice were harbored in the specific pathogen-free facility in Nanjing Medical University. The animals were maintained with free access to pellet food and water in plastic cages at 21 ± 2°C and kept on a 12 h light–dark cycle. To evaluate the lipid metabolic reaction and related genetic changes in the MPTP/p PD models, mice were randomly divided into two groups (*n* = 18/group) for subsequent model induction. The chronic MPTP intoxication protocol was similar to that described previously (Zhou et al., 2016), control mice were treated with saline. All animals were sacrificed 1 week after the final injection. At the endpoint, mice were anesthetized by sodium pentobarbital (50 mg/kg, i.p.), then perfused and sectioned, followed by western blotting (*n* = 6/group), IHC staining (*n* = 6/group), TEM (*n* = 3/group) and RNA-seq (*n* = 3/group) analyses as shown in the schedule (**Figure 1A**) with details showed in respective method sections. For the culture of primary midbrain neurons, pregnant mice with embryonic (E15-17) fetuses were used. The study was approved by the Animal Ethical and Welfare Committee of Nanjing Medical University. All animal welfare and experimental procedures were performed in accordance with the Guide for the Care and Use of Laboratory Animals (National Institutes of Health, United States) and the related ethical regulations of Nanjing Medical University. And all efforts were made to reduce the number of animals used and to minimize animal suffering.

### Cell Culture

Authenticated SH-SY5Y cell lines were cultured in Dulbecco’s Modified Eagle Medium (DMEM, 8115192, 32016001) with 10% FBS (Gibco, Cat 10100147) and 1% penicillin-streptomycin. Cells were cultured to a confluency of 70–80% in six-well or 24-well dishes and transfected with 1 μg of *Plin4* siRNA or NC siRNA (100 nM, Santa Cruz, CA, United States) in OptiMEM using Lipofectamine^TM^ 3000 Transfection Reagent (Invitrogen, Cat L3000-015) for 6 h. For the induction of neuronal damage, cells were incubated with ultrapure MPP^+^ (Sigma, 200 μM) for 24 h. For pharmacological measurements, the autophagy inhibitor 3-MA (Sigma, Cat M-9281, 5 mM) was added to the cell culture medium 1 h before siRNA transfection. The cell or mitochondrial extracts were analyzed by Western blotting.

Primary mesencephalic neurons were obtained and cultured according to our previously described protocol (Xie et al., 2010). After siRNA transfection and incubation with 50 μM MPP^+^ for 48 h, cells were rinsed carefully with PBS and fixed, followed by immunocytochemistry. As to quantification, the number of TH-ir neurons was counted in 10 randomly selected fields on a Nikon Optical TE2000-S inverted microscope. The values were normalized to that obtained from control cultures. The average number of TH-ir cells in control cultures ranged from 20 to 30 per field, with TH-ir cells making up approximately 5% of all cells in the primary culture. Each TH-ir cell process was traced from the soma to the end of the process and quantified by the measurement function of Image-Pro Plus 6.0.

### Induction of the PD Model and Unbiased Stereology

The protocols for generating the MPTP/p-induced chronic PD mouse model and for unbiased stereology have been reported previously (Lu et al., 2014). Briefly, 20 mg/kg MPTP (Sigma, Cat M-0896) dissolved in saline was injected subcutaneously followed by 250 mg/kg DMSO-dissolved probenecid, which blocks the rapid clearance of the neurotoxin MPTP, injection intraperitoneally at 1 h interval every 3.5 days over a period of 5 weeks. Control mice were treated with saline and probenecid. At the endpoint, all animals were anesthetized and perfused, and brains were sectioned for Western blotting and IHC staining. For *in vivo* cell quantification studies, the number of TH^+^ neurons and Nissl^+^ neurons in the SNpc of the midbrain was assessed using the optical fractionator (Stereo Investigator 7, MBF Bioscience, Williston, VT, United States) as previously reported (Lu et al., 2014). All stereological analyses were performed under the 200× objective of an Olympus BX52 microscope (Olympus America Inc., Melville, NY, United States). The stereology was blinded to all genotype and treatment groups for each experiment.

### Tissue Staining, Imaging and Quantification

For frozen samples, mice were perfused transcardially with 4% paraformaldehyde. Brains were extracted, post-fixed, dehydrated, embedded in OCT (Tissue-Tek), and cryosectioned at 30 μm per slice. For immunofluorescence, slides were incubated with the indicated primary antibodies at 4°C overnight, then washed and incubated in secondary fluorescent antibodies, followed by mounting in Prolong Gold Antifade with DAPI (Life Technologies, Cat P36931) before imaging. Images were observed and photographs were captured under a confocal microscope (Axiovert LSM510, Carl Zeiss Co., Germany). The integrated optical densities (IODs) were calculated using ImageJ by sampling of a 30 × 30 pixel area, and 36 images were captured from six consecutive mesencephalon sections. The values were reported as the average intensity above the background ± SD.

For Oil Red O staining, a working Oil Red O solution was generated by diluting a 3.5 mg/ml stock (in 100% isopropanol) (Sigma, Cat 0625) 3:2 with distilled water. This solution was incubated at room temperature for 30 min and filtered with Whatman paper before use. Sections were incubated in 60% isopropanol for 2 min, dried, and incubated in Oil Red O staining solution for 1 h at room temperature. Slides were rinsed in distilled water and counterstained with hematoxylin prior to mounting on Prolong glass slides. For LD staining in CNS sections, slides were submerged in PBS for 10 min and then incubated for 10 min in BODIPY^493/503^ (Life Technologies, Cat D3922). The slides were then washed in PBS and immediately covered with Vectashield mounting medium with DAPI for later imaging on the same day. The LD staining signals were quantitatively analyzed using ImageJ as described above for the immunostaining signals.

### Cell Staining With BODIPY^493/503^ and MitoTracker Deep Red

Live cells were washed twice in PBS and incubated with 2 μg/ml BODIPY^493/503^ (Life Technologies, Cat D3922) in PBS for 15 min at 37°C. For MitoTracker Deep Red staining, live cells were incubated with 0.5 μg/ml MitoTracker Deep Red (Invitrogen, Cat M22426) in PBS for 30 min at 37°C. After staining, the cells were washed in PBS and fixed in 3.5% PFA for 10 min. Then, the cells were washed and counterstained with Hoechst 33342 (Sigma, Cat B2261) for 10 min before being covered on glass slides for imaging. Images were observed and photographs were captured under an optical inverted fluorescence microscope (Nikon, TE2000-S).

### Flow Cytometry Analysis

Mitochondrial membrane potential was measured by fluorescence levels upon staining with JC-1 (Invitrogen, Cat M7514) and MitoTracker Deep Red (Invitrogen, Cat M22426) at 0.5 μg/ml for 30 min at 37°C according to the manufacturer’s instructions. Apoptosis of cells was assessed by staining cells with Annexin V/PI (Invitrogen, Cat V13242) at 37°C for 30 min according to the manufacturer’s instructions. The cells were then washed with PBS and resuspended in cold PBS containing 1% FBS for flow cytometric analyses with Guava easyCyte System 8 (Millipore 25801, Hayward, CA, United States).

### RNA-seq Analysis

Transcriptional profiling via RNA-seq analysis was conducted using the Illumina Hiseq kit according to the manufacturer’s instructions. HTSeq v0.6.1 was used for quantification of gene expression level. Differential expression analysis of the two groups was performed using the DESeq R package (1.10.1). In detail, DESeq provide statistical routines for determining differential expression in digital gene expression data using a model based on the negative binomial distribution. The resulting *P*-values were adjusted using the Benjamini and Hochberg’s approach for controlling the False Discovery Rate (FDR). Genes with an adjusted *P*-value < 0.05 found by DESeq were assigned as differentially expressed. As a result, a particular subset of 74 genes was generated as differentially expressed, with the summary statistical data shown in Supplementary Materials.

### RNA Reverse-Transcription and Quantitative RT-PCR Analysis

Total RNA was extracted with Trizol reagent (Invitrogen Life Technologies, Carlsbad, CA, United States). Total RNA (1 μg) of each sample was reverse-transcribed into cDNA and amplified using a PrimeScript^TM^ RT Master Mix (Takara, RR036A, Takara Biotechnology, China) according to the manufacturer’s directions. RT-PCR was measured using a QuantiTect SYBR^®^ Green PCR kit (Qiagen, Germany) with an ABI 7300 StepOne^TM^ Fast Real-Time PCR System (Applied Biosystems, Foster City, CA, United States). The primer sequences used in this study are listed in the Supplementary Data. After the addition of primers and template DNA to the master, PCR thermal cycle parameters were as follows: 95°C for 3 min, 40 cycles of 60°C for 30 s and 95°C for 15 s, and a melting curve from 60 to 95°C to ensure amplification of a single product. In each sample the *GAPDH* gene was used as an endogenous control to normalize for differences in the amount of total RNA.

### Western Blot, Transmission Electron Microscopy Analysis and Hoechst Staining

The protocols for the Western blot and TEM assays have been reported previously (Lu et al., 2014; Jia et al., 2017). Midbrain and cell protein lysates were quantified by Bradford assays (Bio-Rad, Hercules, CA, United States). Proteins were electrophoresed through a 10–15% SDS–polyacrylamide gel and transferred to PVDF membrane (Millipore, IPVH00010). The membranes were probed with the indicated primary antibodies followed by HRP-conjugated secondary antibodies. Signals were detected by enhanced chemiluminescence (ECL) Western blot detection reagents (Pierce, Rockford, IL, United States). The membranes were scanned and analyzed using an Image Quant LAS 4000 Imaging System (GE Healthcare, Stockholm, Sweden). The average blot intensities were calculated using ImageJ, and the values are reported as the average intensity above the background with β-actin used for normalization.

### Antibody Details

#### Primary Antibodies

Plin4 (Novus, Cat 13776,1:500 for IF &1:1000 for WB); TH (Abcam, Cat ab6211,1:800 for IF); GFAP (Millipore, Cat MAB360,1:500 for IF); poly-Ub (CST, Cat 3936,1:500 for IF & 1:1000 for WB); Tom20 (CST, Cat 13929s,1:500 for IF & 1:1000 for WB);LC3B (CST, Cat 2775,1:500 for IF & 1:1000 for WB); p62 (CST, Cat 5114,1:500 for IF & 1:1000 for WB); Parkin(Abcam, Cat ab15954,1:800 for WB), Cytochrome C (CST, Cat 4272s,1:800 for WB), AIF(E-1) (Santa Cruz, Cat sc-13116,1:800 for WB), caspase-3 (CST, Cat 9662s,1:500 for WB), Beclin-1(CST, Cat 3738,1:1000 for WB), ATG7 (ABGENT, Cat AP1813a,1:800 for WB), β-actin (Santa Cruz, Cat SC-47778,1:1000 for WB). Secondary antibodies: Alexa F488 Donkey anti-mouse (Invitrogen, Cat A21202, 1:1000), Alexa Fluor 488 Goat anti-Rabbit (Invitrogen, Cat A11008,1:1000), Alexa Fluor 594 Donkey anti-Goat (Invitrogen, Cat A11058, A11008, A 11059,1:1000), HRP-conjugated secondary antibodies (Thermo, Cat 0031430, 31460, 31402, 1:1000).

### Statistical Analysis

All data are presented as the means ± SEM and were collected and analyzed in a blinded manner. Statistical analysis was performed using Student’s *t*-test or one-way analysis of variance (ANOVA) followed by the Holm–Sidak test (SigmaPlot 11.0). Two-way ANOVA was used when the genotype and treatment were considered as two independent variables. The tests used are indicated in the figure legends. In all studies, n indicates the number of samples per group, and cases in which *P-*values < 0.05 were considered statistically significant.

## Results

### LDs Accumulate in the Mesencephalon of MPTP/p-Treated Mice

Emerging studies have documented unexpected LD accumulation accompanied by mitochondrial dysfunction that promotes neurodegeneration in Drosophila (Liu et al., 2015). To determine whether LD formation occurs in mammalian models and participates in the pathogenesis of PD, we generated the MPTP neurotoxin-induced PD mouse model for chronic mitochondrial dysfunction-associated loss of DA neurons using a previously described protocol (Lu et al., 2014). As shown by IHC staining, PD model mice were well established, with both TH^+^ and Nissl^+^ neurons in the SNpc showing a dramatic reduction in number (41.5 and 52.5%, respectively) in MPTP-treated mice compared with those in the saline-injected control group (**Figures 1B,C**).

Transmission electron microscopy indicated that MPTP injection induced a significant accumulation of LDs in the cytosol of neurons (indicated by red stars; morphologically seen with low and homogeneous electron density, little heterochromatin, and a large, round nucleolus for neuronal cell identification). LDs are easily identifiable in **Figure 1D**, similar to results reported previously (Singh et al., 2009), showing round, light-density structures not limited by a lipid bilayer membrane (Middle frame, red arrow), with homogenous amorphous content that was increased in both size and amount in response to MPTP/p stimuli. In contrast, LDs were rarely observed or were found to have a much smaller size in controls. Moreover, MPTP/p-induced LD accumulation was accompanied by mitochondrial damage (Right frame) and nearby autolysosomes (middle frame, yellow arrow). These findings suggest abnormal LD deposition in SNpc neurons that may correlate with mitochondrial damage in this PD model. For further confirmation, we performed LD-specific staining in brain sections and observed LD deposition (enhanced neutral lipid) in both the SNpc and striatum by using BODIPY^493/503^ (**Figure 1E**) and Oil Red O (**Figure 1F**). LD-specific staining was especially dense in both the SNpc and striatum of injured animals, indicating much more LD accumulation in regions where the bodies and axons of TH-ir neurons are located, whereas control animals showed diffuse staining (**Figures 1E,F**). These results suggested MPTP/p toxicity could induce mitochondrial damage with concomitant LD accumulation in the midbrain neurons of PD model mice.

### Increased Plin4 Expression Is Positively Correlated With LD Accumulation in the Midbrain of MPTP/p-Treated Mice

**FIGURE 2:**
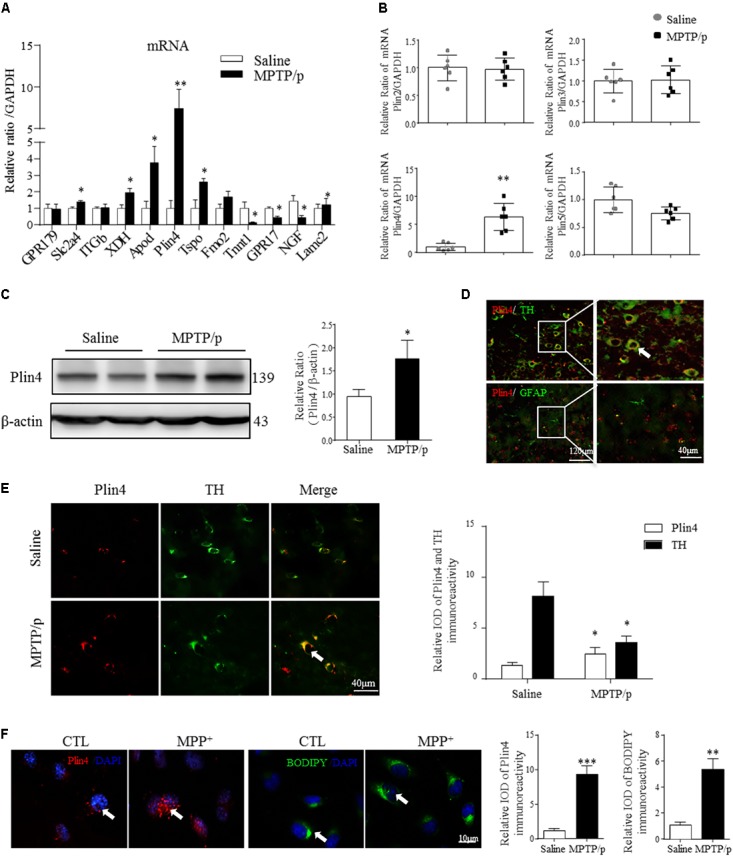
Increased Plin4 is positively correlated with LD accumulation in the midbrain of MPTP/p-treated mice. **(A,B)** A: Quantitative RT-PCR analysis of RNA-seq indicated increased genes. B: RT-PCR analysis of Plin4 and other perilipins in mesencephalon samples from matched PD and NC mice. **(C)** Western blot analysis was performed to assess Plin4 expression *in vivo.* **(D,E)** For cellular location, the expression of Plin4 and TH/GFAP were analyzed by immunofluorescence of frozen brain sections. Arrows indicate neurons with coexpression of Plin4 and TH; the integrated optical density (IOD) of Plin4 and TH staining is presented on the right. **(F)** SH-SY5Y cells were stimulated with MPP^+^ for 24 h followed by IF of Plin4 and BODIPY^493/503^ staining (arrows marked), quantitation showed in right. Scale bar as indicated. Data are presented as the means ± SEM. Data in **(A,E)**: ^∗^*P* < 0.05, ^∗∗^*P* < 0.01 vs. the control group as determined by one-way ANOVA (*n* = 4). Data in **(B,C,F)**: ^∗^*P* < 0.05, ^∗∗^*P* < 0.01, ^∗∗∗^*P* < 0.001 vs. the control group as determined by Student’s *t*-test (*n* = 6 in **B,C**).

Lipid droplets functions are regulated predominantly by the surrounding perilipin proteins of LDs (Kimmel and Sztalryd, 2016). To confirm whether and which perilipin may contribute to LD accumulation in the PD model mouse brain, we conducted the RNA-seq and subsequent quantitative PCR analysis of mesencephalon samples from saline- and MPTP/p-challenged mice. As quantitative PCR revealed, among all the increased genes (*P*-value < 0.05, details in Supplemental Materials), *Plin4* showed the highest overexpression in MPTP/p-treated mouse brain (**Figure 2A**). In contrast, the mRNA level of other perilipins was not elevated (**Figure 2B**). We also found a much higher *Plin4* level in midbrain and striatum, compared to that in cortex and hippocampus (Supplementary Figure S1), further supporting the relationship between *Plin4* and PD. Correspondingly, Western blotting also revealed a significant upregulation of Plin4 protein in the midbrain of PD model mice (**Figure 2C**). As shown in **Figure 2D**, co-immunostaining of Plin4 and different cell markers demonstrated that Plin4 mainly localized in TH^+^ neurons but not in GFAP^+^ glia. Indeed, MPTP/p challenge-induced elevation of Plin4 expression was further confirmed by IHF (**Figure 2E**), supporting the evidence that the LD-related Plin4 change indeed occurred in DA neurons. To confirm this result *in vitro*, SH-SY5Y cells, a representative cell line of dopaminergic neurons, were cultured and then stimulated with MPP^+^ to mimic MPTP/p-induced neurological damage *in vivo*. Consistent with the *in vivo* findings, MPP^+^ treatment promoted Plin4 expression and intracellular neutral lipid deposition as determined by BODIPY^493/503^ staining (**Figure 2F**) in SH-SY5 cells. Together, these findings indicate increased Plin4 expression, rather than that of other perilipins, may account for LD accumulation and participate in PD pathogenesis.

### Knockdown of *Plin4* Decreases LD Accumulation and Alleviates SH-SY5Y Cell Damage

**FIGURE 3:**
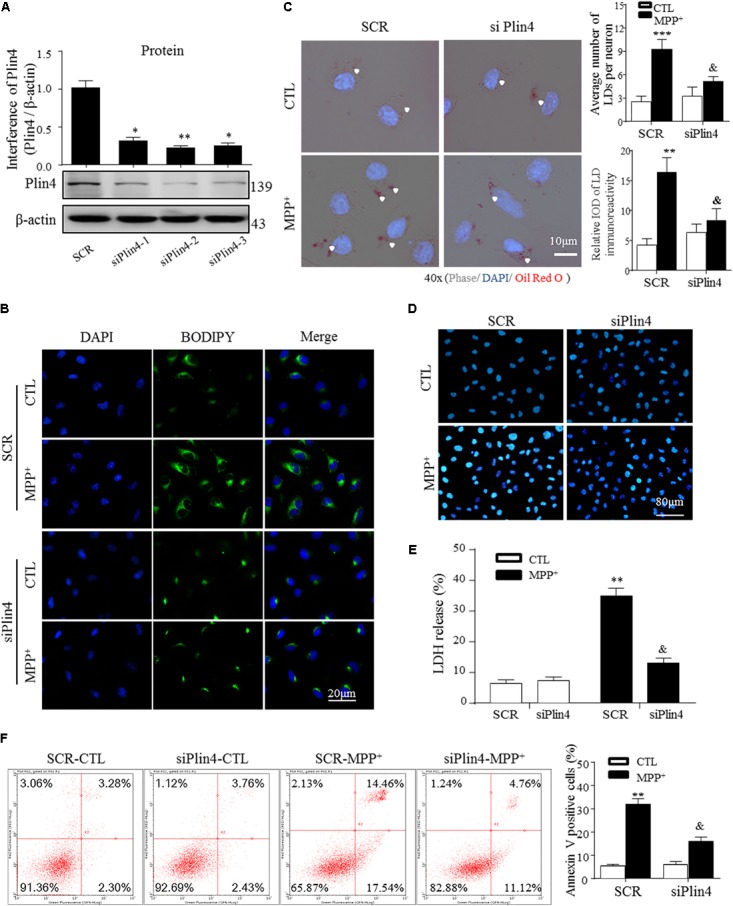
Knockdown of *Plin4* decreases LD accumulation and alleviates SH-SY5Y cell damage. **(A)** SH-SY5Y cells were transduced with siRNA against *Plin4* or an SCR control. Western blot analysis was performed at 48 h post-transduction to assess for Plin4 suppression. ^∗∗^*P* < 0.01, ^∗∗∗^*P* < 0.001 vs. SRC as determined by one-way ANOVA followed by the Holm–Sidak test. **(B)** Cells described in **(A)** followed by CTL (PBS alone) or 200 μM MPP^+^ stimulation for another 24 h. Live cells were stained with BODIPY^493/503^ (2 μg/ml) then fixed and counterstaining with DAPI for fluorescence imaging. **(C)** Oil Red O staining of the cells described in **(B)**. Right: quantitation of LD number and integrated optical density (IOD). **(D)** *Plin4* knockdown and MPP^+^ stimulation-induced cytotoxicity in SH-SY5Y cells were determined by Hoechst 33342 staining. **(E)** The cells described in **(B)** were subjected to the LDH release assay after 24 h of treatment. **(F)** Annexin V/PI staining indicated apoptosis of the cells described in **(E)**. Data in **(C,E,F)** are shown as mean ± SEM. ^∗^*P* < 0.05, ^∗∗^*P* < 0.01 vs. SCR-CTL, ^&^*P* < 0.05 vs. SCR-MPP^+^ as determined by two-way ANOVA.

To directly examine the role of Plin4 in LD accumulation due to MPP^+^ stimuli *in vitro*, we transfected multiple siRNAs targeting *Plin4* or a scrambled control (SCR) into SH-SY5Y cell lines and observed that both *Plin4* mRNA (Supplementary Figure S2) and protein expression were decreased (**Figure 3A**). LDH release assay and Hoechst 33342 staining both indicated that the *Plin4* knockdown alone had no effect on cell viability (**Figures 3D,E**). By staining with the lipid-specific indicator Oil Red O and BODIPY^493/503^, we further revealed that *Plin4* silencing indeed reversed MPP^+^-induced intracellular LD accumulation (**Figures 3B,C**). Furthermore, to assess the functional capacity of LDs, SH-SY5Y cells were transfected with *siPlin4* or SCR plus MPP^+^ treatment, and we found that Plin4 depleted cells were more tolerant to MPP^+^ induced toxicity, evidenced by both Hoechst 33342 staining and LDH release assay (**Figures 3D,E**), consistent with reduced lipid storage within LDs. In addition to the Annexin V/PI staining indicated, the percentage of apoptotic cells (**Figure 3F**) further confirmed that cell damage was alleviated by *Plin4* silencing. Collectively, these results showed that *Plin4* inhibition decreased MPP^+^-induced LD deposition and enhanced cell viability. Thus, *Plin4* is essential for LD deposition and accounts for cell damage upon toxic mitochondrial insult.

### Knockdown of *Plin4* Reverses Mitochondrial Damage in the MPP^+^ Cell Model

**FIGURE 4:**
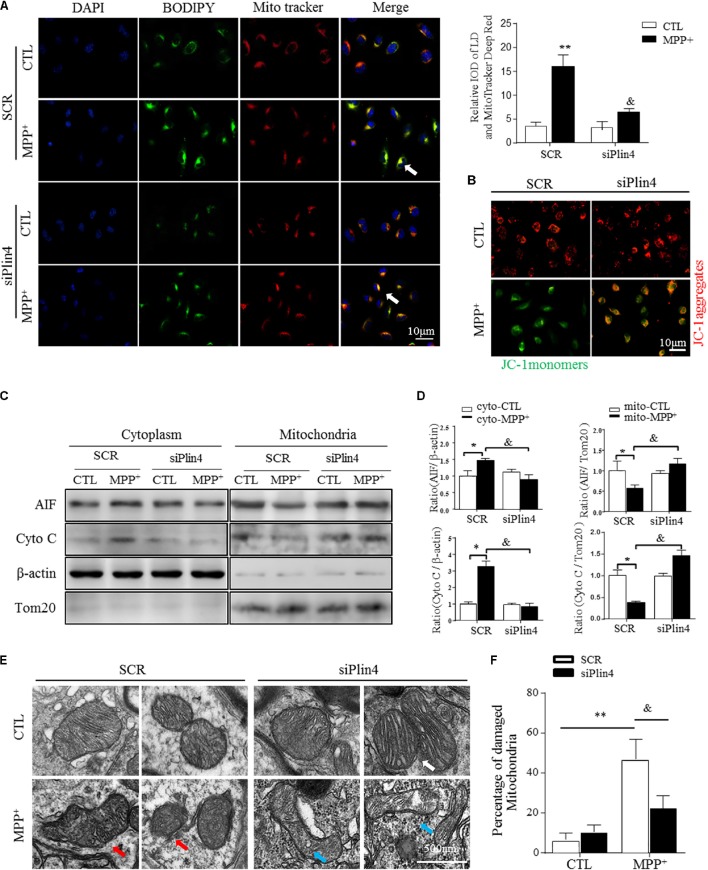
Knockdown of *Plin4* reverses mitochondrial damage in MPP^+^ cell model. **(A)** Intracellular distribution of LDs (BODIPY^493/503^) and mitochondria (MitoTracker Deep Red) in MPP^+^-stimulated SH-SY5Y cells were examined by fluorescence microscopy. Arrows indicate cells with colocalization of LDs and mitochondria; the integrated optical density (IOD) of BODIPY^493/503^ and MitoTracker Deep Red immunofluorescence is presented on the right. **(B)** MPP^+^-induced mitochondrial membrane potential changes in WT (SCR) or *Plin4*-deficient (siPlin4) SH-SY5Y cells were measured by JC-1 staining. Scale bar: 10 μm. **(C)** SH-SY5Y cells described in **(B)** were harvested, and then, mitochondria and cytosol were separated using a commercial kit and assessed for AIF and Cyto C expression. **(D)** Quantification of protein expression in **(C)**. β-actin and Tom20 were utilized as endogenous control genes for cytosol and mitochondria, respectively, and relative expression levels were determined by normalizing to those in the SCR-CTL samples. **(E)** Electron micrographs of mitochondria in WT (SCR) or *Plin4-*deficient (siPlin4) SH-SY5Y cells incubated with MPP^+^ as described above. Shown are representative examples of normal (white arrow), partially damaged (blue arrow), and heavily damaged mitochondria (red arrow). Scale bar: 500 nm. **(F)** Quantification of damaged mitochondria in **(E)**. Data in **(A,D,F)** are shown as mean ± SEM. ^∗^*P* < 0.05, ^∗∗^*P* < 0.01 vs. SCR-CTL, ^&^*P* < 0.05 vs. SCR-MPP^+^ as determined by two-way ANOVA.

It has been reported that altered LDs could trigger dysfunction of many intracellular organelles, especially the mitochondria (Welte, 2015), and that this is mainly mediated by the surface coated protein, perilipin. Thus, we proposed that Plin4-mediated LD deposition could magnify mitochondrial stress, contributing to subsequent death-signals being released and damage initiated. To confirm this, we first examined whether Plin4 affects the subcellular distribution of LDs and/or leads to mitochondrial translocation by MPP^+^ stimuli. As showed in **Figure 4A**, LDs displayed rare and diffuse cytoplasmic distribution in PBS-treated controls. In contrast, MPP^+^ incubation caused LD deposition and localization with or adjacent to, damaged mitochondria. Moreover, *Plin4* deficiency could prevent this MPP^+^-induced LD shifting. Then, to assess the functional association between *Plin4* and mitochondrial damage, the mitochondrial membrane potential was measured by JC-1 staining in SH-SY5Y cells. As **Figure 4B** indicated, the decrease in membrane potential induced by MPP^+^ was alleviated by *Plin4* silencing. We also examined the expression of apoptosis-inducing factor (AIF) and Cytochrome C (cyto C), both of which translocate from the mitochondria into the cytoplasm in the early phases of mitochondrial damage to initiate the apoptotic proteolytic cascade by activating caspase-3. Western blotting showed more AIF and cyto C released into the cytoplasm upon MPP^+^ stimulation (**Figures 4C,D**), which was partly reversed by *Plin4* deletion. For direct mitochondrial integrity assessment, we further observed the ultramicromorphological changes in SH-SY5Y cells. MPP^+^ triggered accumulation of numerous swollen mitochondria in the SCR control cells, containing many highly damaged, electron-dense disrupted cristae mitochondria, which was attenuated by *Plin4* knockdown (**Figures 4E,F**). These results indicate that genetic silencing of *Plin4* alleviated MPP^+^-induced mitochondrial damage in a dopaminergic cell line.

### Downregulation of *Plin4* Activates Mitophagy in MPP^+^-Treated SH-SY5Y Cells

**FIGURE 5:**
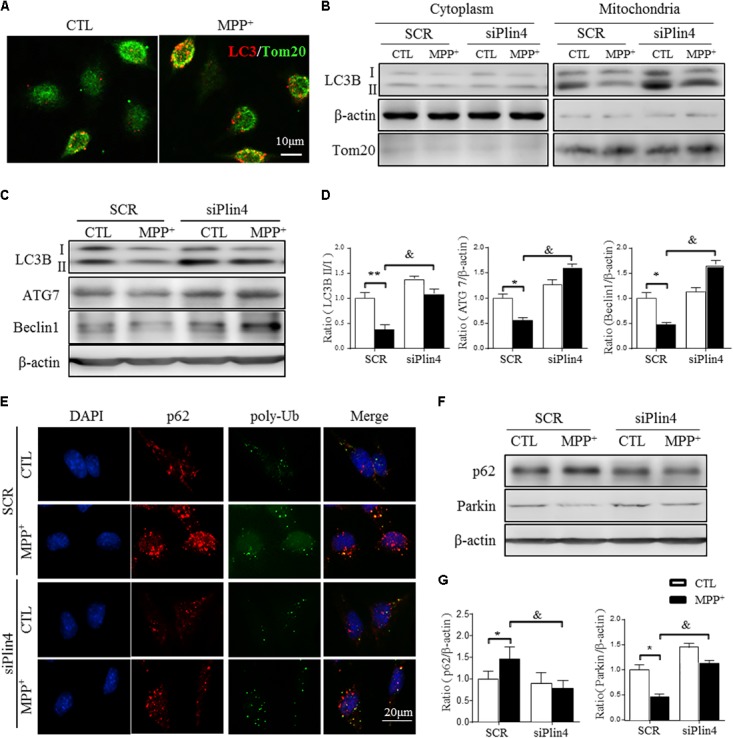
Downregulation of *Plin4* activates autophagy in MPP^+^-treated SH-SY5Y cells. **(A)** SH-SY5Y cells were treated with 200 μM MPP^+^ for 24 h. LC3-II dot localization was detected by ICF with Tom20 as a mitochondrial marker. Scale bar: 10 μm. **(B)** SH-SY5Y cells were transfected with SCR or siPlin4 for 24 h, followed by MPP^+^ incubation (200 μM, 24 h). Mitochondria and cytosol were then separated and assessed for LC3B expression. **(C,D)** C: Cells treated as in **(B)** were harvested, and proteins were collected for detection of LC3B, ATG7, and Beclin1 expression, with quantification shown in **(D)**. **(E)** Cells described in **(B)** were fixed, followed by ICF staining of p62 and poly-Ub sequestration and intracellular localization. Scale bar: 20 μm. **(F,G)** Cells described in **(C)** were harvested for detection of p62 and Parkin expression and relative quantification. Data in **(D,G)** are shown as the mean ± SEM. ^∗^*P* < 0.05, ^∗∗^*P* < 0.01 vs. SCR-CTL, ^&^*P* < 0.05 vs. SCR-MPP^+^ as determined by two-way ANOVA.

Mitophagy, a quality control process, mediates the clearance of damaged ubiquitinated mitochondria, and mutations in genes related to mitophagic function, especially PINK1 and DJ-1, account for many autosomal recessive forms and some sporadic cases of PD (Lazarou et al., 2015). As shown in **Figure 5A**, MPP^+^ induced an increase in LC3-labeled vacuoles (namely, foci of LC3-II, the autophagosomal marker microtubule-associated protein 1 light chain 3) with a localization adjacent to mitochondria (as indicated by Tom20, a mitochondrial membrane protein) in SH-SY5Y cells. To detect the participation of *Plin4* in mitophagy inhibition, we separated mitochondria from the cytoplasm and examined the expression of MAP1LC3B/LC3B-II, which is a cleaved MAP1LC3B-phosphatidylethanolamine conjugate and a general autophagosomal marker. We showed that Plin4 knockdown promoted mitophagy, as evidenced by more conversion of LC3-I to LC3-II in the mitochondria, which was previously inhibited by MPP^+^ stimulation (**Figure 5B**). To further characterize the underlying autophagic mechanisms of *Plin4* in mitophagy inhibition, we further analyzed the expression of many autophagy-related proteins in mitochondria. As well as LC3B-II production, *Plin4* silencing also restored MPP^+^-induced ATG7 and beclin1 inhibition (**Figures 5C,D**). These results indicated that MPP^+^ blunted mitophagy could be rescued by *Plin4* silencing in SH-SY5Y cells.

During the autophagic process, p62 acts as a receptor protein that links LC3B with ubiquitinated substrates for clearance (Komatsu et al., 2012). Consistently, we found that MPP^+^-induced p62 and poly-Ub sequestration was reduced by *Plin4* deficiency, as evidenced by both immunofluorescence (**Figure 5E**) and Western blotting (**Figures 5F,G**). Parkin, an identified mitophagy-related E3 ligase (Lazarou et al., 2015; Pickrell and Youle, 2015), is a key mediator of this p62-Ub mediated clearance of damaged mitochondrial and was also reversed by Plin4 deficiency (**Figures 5F,G**). Collectively, these results imply that inhibition of Plin4 promoted mitochondrial homeostasis by activating autophagic removal of damaged substrates in the PD cell model.

### 3-MA Abolishes the Protective Effect of *Plin4* Deficiency on Primary DA Neurons

**FIGURE 6:**
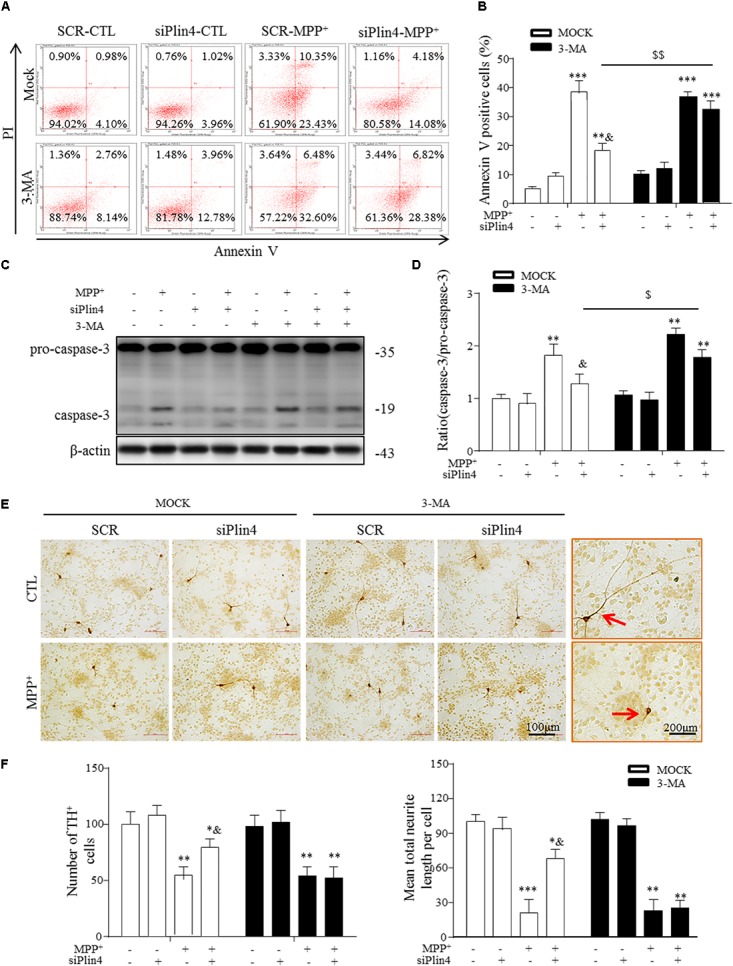
3-MA abolishes the protective effect of Plin4 deficiency on primary DA neurons. **(A,B)** A: SH-SY5Y cells were pretreated with PBS or 3-MA, followed by siRNA transfection 24 h before MPP^+^ was given, and apoptosis was assessed by Annexin V/PI staining and flow cytometry. The data for flow cytometry analysis are presented as a percentage of the cell population by normalizing to MOCK-SCR-CTL samples. **(C,D)** Cells described in **(A)** were harvested and proteins were collected for the detection of caspase-3 activity by Western blot. **(E,F)** E: Primary cultured neurons of the mesencephalon suffered indicated treatments, followed by TH IHC detection; amplifications shown in the right frames for details. F: Representative number of TH^+^ neurons and neurite length compared with SCR-NC. Scale bar as indicated. Data in **(B,D,F)** are shown as the mean ± SEM (*n* = 4–6). ^∗^*P* < 0.05, ^∗∗^*P* < 0.01, and ^∗∗∗^*P* < 0.001 vs. SCR-NC-CTL; ^&^*P* < 0.05 vs. SCR-NC-MPP^+^; and ^$^*P* < 0.05,^$$^*P* < 0.01 vs. MOCK-NC-MPP^+^ as analyzed by one-way ANOVA followed by the Holm–Sidak test.

Damaged mitochondria activate multiple signals, such as mitochondrial reactive oxygen species (mtROS), mtDNA and cardiolipin, promoting the mitochondrial apoptosis pathway, which represents the primary reason for the loss of TH-ir neurons in PD (Suliman and Piantadosi, 2016; Dawson and Dawson, 2017). Because our data indicate that Plin4 hindered autophagy, which accounted for MPP^+^-induced mitochondrial damage, we speculated that promotion of the autophagic elimination of these danger signals by controlling *Plin4* might attenuate injuries triggered by MPTP toxicity. Therefore, we treated NC and Plin4-silenced SH-SY5Y cells with 3-MA, an autophagy inhibitor that can block the maturation and degradation of autophagy. Flow cytometry results using Annexin V/PI indicated that 3-MA pretreatment abolished the cell death-reducing effect of *Plin4* knockdown in MPP^+^-stimulated SH-SY5Y cells (**Figures 6A,B**). These results were confirmed by immunoblot analysis of activation of caspase-3 (**Figures 6C,D**), an apoptosis-related protein that is proteolytically activated upon exposure to apoptotic stimuli. To provide more direct evidence linking DA neuronal deterioration, mitophagy and *Plin4*, primary mesencephalic neurons were cultured as described previously (Qiao et al., 2016), followed by MPP^+^ stimulation to mimic the toxicity of MPTP/p *in vivo*. As shown in **Figures 6E,F**, MPP^+^ decreased the number of TH-ir neurons by 43.4% and shortened neurite length by 65.7% compared with those in controls. In agreement with the results obtained in SH-SY5Y cells, MPP^+^-induced damage in primary TH-ir neurons was also partially alleviated by siPlin4 transfection. Moreover, the neuroprotection contributed by Plin4 silencing was suppressed by 3-MA, supporting the idea that autophagy was involved in the *Plin4-*mediated deterioration of DA neurons.

## Discussion

The accumulation of lipid metabolites in nonadipose tissues causes lipotoxicity (Wymann and Schneiter, 2008) and is correlated with insulin resistance, type II diabetes mellitus, hepatic steatosis, and cardiovascular disease (Wymann and Schneiter, 2008; Gan et al., 2014; Schaffer, 2016). Lipotoxicity was found clinically relevant with NDDs, (Aviles-Olmos et al., 2013; Zou et al., 2015; Ascherio and Schwarzschild, 2016), although clear links have not been elucidated. Inside cells, lipids are stored within micelles known as LDs. Emerging evidence suggests the development of lipotoxicity is not solely due to the presence of FFAs in nonadipose tissues but is also due to the alteration of LD homeostasis (Krahmer et al., 2013; Thiam and Beller, 2017). Defects in LD biogenesis or turnover lead to perturbance of many metabolic pathways, further causing inflammation and mitochondrial and ER stress (Welte, 2015; Mukhopadhyay et al., 2017). Clinically, new evidence has emphasized the importance of LDs in cancers and aging-related diseases, as well as nervous system homeostasis (Bailey et al., 2015; Liu et al., 2015; Qiu et al., 2015; Conte et al., 2016). Recently, Liu et al. (2015) revealed that mutations in mitochondrial-related genes exhibit a common phenotype of LD accumulation, promoting the onset of neurodegeneration (ND) in Drosophila (Liu et al., 2015). However, a direct link between LD accumulation and NDD in mammals has not been established thus far.

In this study, via TEM and lipid-specific staining, we showed that MPTP/p-treated PD model mice exhibited LD accumulation in exactly the same brain region where DA neuronal loss occurs. Bailey et al. reported similar LD changes occurred in glial cells to preserve neuroblast proliferation under hypoxic conditions in Drosophila (Bailey et al., 2015). In our mouse model, both TEM and IHF co-immunostaining of TH/GFAP with Plin4 consistently revealed that LDs mainly clustered in neurons, with less accumulation in astrocytes. Whether LDs may perform some glial-specific function requires further exploration. Thus, our *in vivo* study suggests a strong correlation between LD deposition and mitochondrial stress in a specific MPTP/p-induced mouse model of PD.

Our results fit within an emerging theme in which primary injuries coordinately induce both lipid synthesis processes and a disturbance in mitochondrial homeostasis, which cooperate in reducing cell viability, representing a common change in many energy metabolism-related diseases, including NDDs (Qiu et al., 2015; Thiam and Beller, 2017). Specifically, evidence indicates LD accumulation resulting from mitochondrial dysfunction precedes physical and histological changes in NDD (Liu et al., 2015) and also protects neural stem cells from hypoxia damage during development in Drosophila (Bailey et al., 2015). Thus, in the case of energy barriers predominantly in CNS, LDs, the novel but incompletely defined organelles, may represent the primary and unique way for the organisms to solve the problem of balancing energy supply and cellular homeostasis. However, once the compensation cannot be satisfied or the stimuli sustained, LDs may subsequently change to be harmful and aggravate the injuries by inducing mitochondrial stress, ER stress or autolysosome dysfunction (Welte, 2015), as happened in our study.

Lipid droplets are highly dynamic organelles surrounded by a phospholipid monolayer and several proteins (Welte, 2015). The most abundant and well-characterized LD coating proteins belong to the so-called PAT family, comprising five members (1–5) collectively known as Perilipins (Kimmel and Sztalryd, 2016), which have physiological roles in facilitating storage of neutral lipids within LDs and regulating the intracellular interactions with other organelles. Here, with the combined use of RNA-seq and RT-PCR, we identified upregulated expression of Plin4 accompanied by LD accumulation in the MPTP/p-treated mouse midbrain. Plin4 is normally found decorating nascent LDs in the cytosol, and its inactivation has been reported to affect heart function (Chen et al., 2013). Moreover, genetic silencing of Plin4 significantly ameliorated LD storage and promoted the survival of neurons *in vitro*. Hence, the molecular mechanisms underlying Plin4-controlled LD deposition are likely to be conserved in mice and may contribute to the pathogenesis of PD. The clarification of this question may help to provide novel explanations for the theory of lipotoxicity in PD.

Mitochondrial disability is a common feature of both familial and sporadic PD, as well as toxin-induced parkinsonism (Newmeyer and Ferguson-Miller, 2003; Ryan et al., 2015; Suliman and Piantadosi, 2016). Recent findings have further revealed mitophagy dysfunction to be clinically relevant, with abnormal lipid metabolism being suggested to relate to PD pathogenesis. Here, we showed that Plin4 silencing conferred a reduction in LD deposition and in turn resulted in prevention of MPP^+^-induced mitochondrial damage as evidenced by mitochondrial membrane potential collapse, mitochondrial fragmentation, and mitophagy inhibition. Thus, our findings fit with the newly identified mitochondria-related roles of LDs (Welte, 2015).

In addition to their interactions with mitochondria, LDs also interact with autolysosomes, with several studies demonstrating a bidirectional relationship between the two (Jaishy and Abel, 2016). By examining the linkage between LD disturbance and autophagic signaling, we showed that Plin4 silencing-driven autophagy and p62-ubiqutin mediate the autophagic clearance of damaged mitochondria, which in turn ameliorated deterioration in both SH-SY5Y cells and primary cultured DA neurons. We present our conclusions based on the following observations: Plin4 deficiency conferred apoptosis inhibition and caspase-3 inactivation, which were significantly blocked by the autophagy inhibitor 3-MA.

## Conclusion

In conclusion, by exploring the role of LDs *in vitro* and in a mouse PD model *in vivo*, we demonstrated that Plin4-dependent LD deposition in TH-ir neurons contributed to DA neuronal loss in MPTP/p-treated mice. Mechanistically, excessive accumulation of LDs may trigger mitochondria-impaired mitophagy, further resulting in subsequent neurodegenerative damage. Thus, a dysfunctional Plin4/LD/mitophagy axis is clarified to be involved in PD pathophysiology, indicating that Plin4-LD changes in the brain may be a promising biomarker as well as therapeutic target for PD.

The main limitation of this work is the use of a chemical insult-based model of PD, which cannot mimic the whole spectrum of PD pathogenesis. Replicating these findings in different PD models would help to verify the possibility of rendering Plin4-LDs as therapeutic targets for PD.

## Author Contributions

GH and ML p designed the research. XH performed co-transfection for the western blotting assays, neuropathological study, RT-PCR and drafted the manuscript. JZ performed the immunohistochemistry study and statistical analysis. JZ and SS contributed to the statistical analysis. XZ and QS helped to perform the western blotting assays and contributed materials/analysis tools. JD and SS discussed the project and gave valuable suggestions to this project. All authors read and approved the final manuscript.

## Conflict of Interest Statement

The authors declare that the research was conducted in the absence of any commercial or financial relationships that could be construed as a potential conflict of interest.

## Abbreviations

3-MA: 3-Methyladenine
CNS: central nervous system
ER: endoplasmic reticulum
FFAs: free fatty acids
IHC: immunohistochemistry
LDH: lactate dehydrogenase
LDs: lipid droplets
MPTP: methyl-4-phenyl-1,2,3,6-tetrahydropyridine
NC: negative control
NDDs: neurodegenerative diseases
PD: Parkinson’s disease
poly-Ub: poly-ubiquitin
SNpc: substantia nigra pars compacta
TEM: transmission electron microscopy
TH: tyrosine hydroxylase.
