# Supplementary material for: Plin4-Dependent Lipid Droplets Hamper Neuronal Mitophagy in the MPTP/p-Induced Mouse Model of Parkinson’s Disease
Source: Front Neurosci. 2018 Jun 18;12:397. doi: 10.3389/fnins.2018.00397 (PMC6015897; doi:10.3389/fnins.2018.00397)

**Supplemental Materials 2-1:**

Supplemental figures:


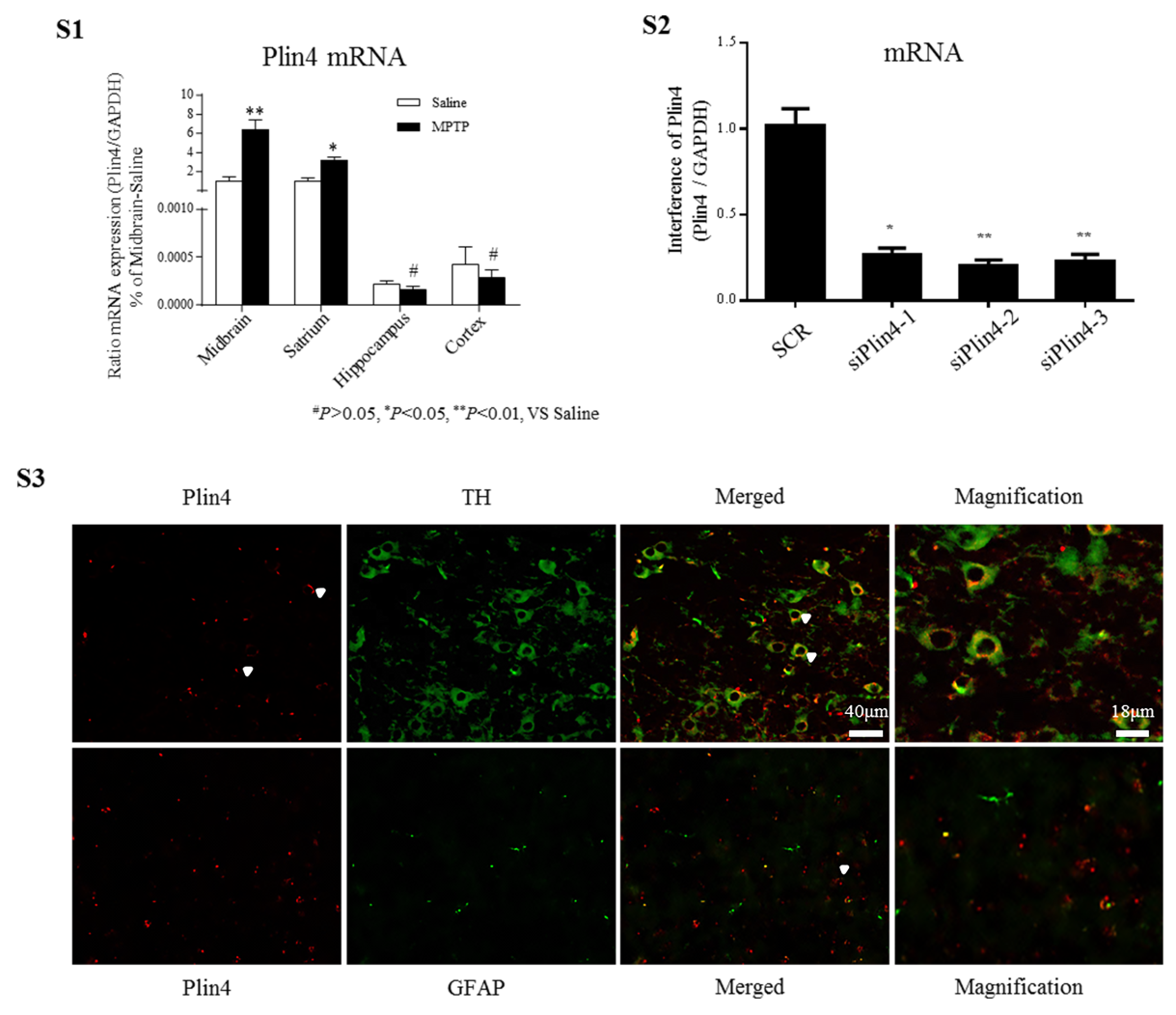


**Figure S1.** RT-PCR analysis of Plin4 in different brain region of Saline and MPTP/p mice. ^*^P<0.05, ^**^*P*<0.01, vs. Saline group analyzed by one-way ANOVA.

**Figure S2.** RT-PCR analysis of Plin4 in SH-SY5Y cells transduced with siRNA against Plin4 or a SCR control, *GAPDH* were utilized as endogenous control genes and relative mRNA expression was determined by normalizing to expression in SCR samples. ^*^*P*<0.05, ^**^*P*<0.01, vs. SCR. *P*-values were determined by one-way ANOVA followed by the Holm-Sidak test.

The sequences of primers for qPCR analysis are as follows:

*Mouse GAPDH Forward 5’-CAAAAGGGTCATCTCC -3’ ;*

*Mouse GAPDH Reverse 5’-CCCCAGCATCAAAGGTG-3’ ;*

*Mouse Plin2 Forward 5’-TGGTGAGTGGCCTGTGTTAG-3’ ;*

*Mouse Plin2 Reverse 5’- GCACACGCCTTGAGAGAAAC -3’ ;*

*Mouse Plin3 Forward 5’-CAAGGAGAACTACCCCCACG-3’ ;*

*Mouse Plin3 Reverse 5’-GGTCTTGTCCACGCTGTTCT -3’ ;*

*Mouse Plin4 Forward 5’- GACCAGCAGTGAAGATGCCT -3’ ;*

*Mouse Plin4 Reverse 5’-TCCTTCGTATTGGTGAGGACA -3’ ;*

*Mouse Plin5 Forward 5’- CGTGCAATCTTTGCCTCAAC-3 ’ ;*

*Mouse Plin5Reverse 5’- GCCGAGTTGTAAGCACTGGA -3’ ;*

*Human GAPDH Forward 5’-ACATCCATCGCCACCACTAC-3’ ;*

*Human GAPDH Reverse 5’-ATGTGACAGATGGGGCTGAC -3’;*

*Human Plin4 Forward 5’-CATGGACGGAGAAGGAGCTG -3’;*

*Human Plin4 Reverse 5’-CGGTGAGGACAGCCTTCG -3’ .*

Western blotting:


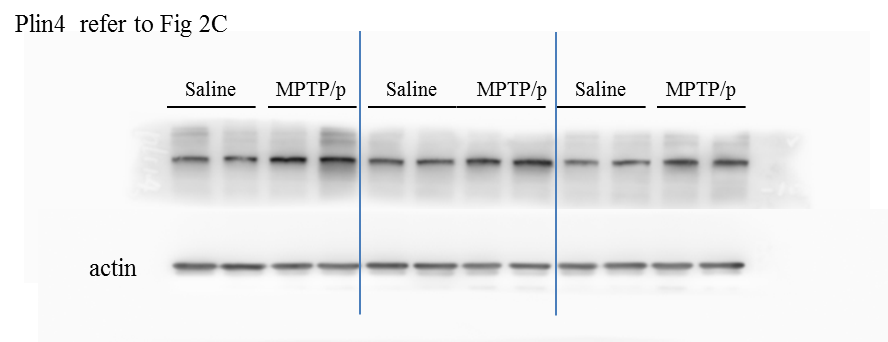

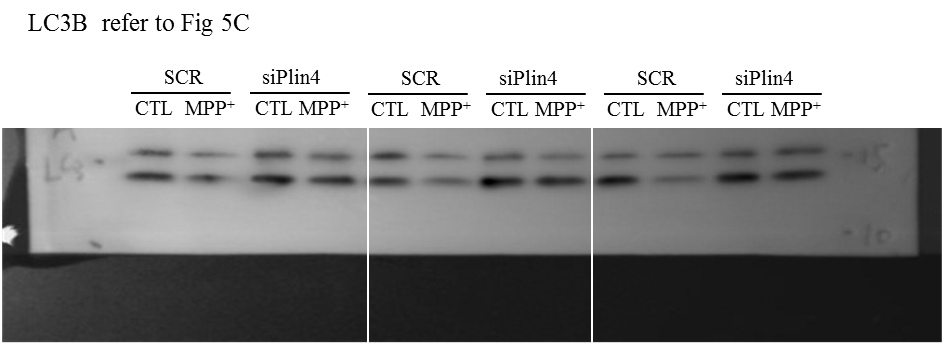

Supplement: Supplementary file 2 [file Data_Sheet_2.DOCX]
